# Site-selectively generated photon emitters in monolayer MoS_2_ via local helium ion irradiation

**DOI:** 10.1038/s41467-019-10632-z

**Published:** 2019-06-21

**Authors:** J. Klein, M. Lorke, M. Florian, F. Sigger, L. Sigl, S. Rey, J. Wierzbowski, J. Cerne, K. Müller, E. Mitterreiter, P. Zimmermann, T. Taniguchi, K. Watanabe, U. Wurstbauer, M. Kaniber, M. Knap, R. Schmidt, J. J. Finley, A. W. Holleitner

**Affiliations:** 1 Walter Schottky Institut and Physik Department, Technische Universität München, Am Coulombwall 4, 85748 Garching, Germany; 2 Nanosystems Initiative Munich (NIM), Schellingstr. 4, 80799 Munich, Germany; 3 Institut für Theoretische Physik, Universität Bremen, P.O. Box 330 440,, 28334 Bremen, Germany; 4 Bremen Center for Computational Materials Science, University of Bremen, Am Fallturm 1, 28359 Bremen, Germany; 5 Department of Physics, University at Buffalo, The State University of New York, Buffalo, NY 14260 USA; 6 National Institute for Materials Science, Tsukuba, Ibaraki 305-0044 Japan; 7 Department of Physics and Institute for Advanced Study, Technical University of Munich, 85748 Garching, Germany; 8 Max-Planck-Institut für Quantenoptik, 85748 Garching, Germany

**Keywords:** Two-dimensional materials, Single photons and quantum effects

## Abstract

Quantum light sources in solid-state systems are of major interest as a basic ingredient for integrated quantum photonic technologies. The ability to tailor quantum emitters via site-selective defect engineering is essential for realizing scalable architectures. However, a major difficulty is that defects need to be controllably positioned within the material. Here, we overcome this challenge by controllably irradiating monolayer MoS_2_ using a sub-nm focused helium ion beam to deterministically create defects. Subsequent encapsulation of the ion exposed MoS_2_ flake with high-quality hBN reveals spectrally narrow emission lines that produce photons in the visible spectral range. Based on ab-initio calculations we interpret these emission lines as stemming from the recombination of highly localized electron–hole complexes at defect states generated by the local helium ion exposure. Our approach to deterministically write optically active defect states in a single transition metal dichalcogenide layer provides a platform for realizing exotic many-body systems, including coupled single-photon sources and interacting exciton lattices that may allow the exploration of Hubbard physics.

## Abstract (web summary)

Light emitters can be induced in transition metal dichalcogenides by defect engineering, but challenges remain in their controlled spatial positioning. Here, the authors irradiate monolayer MoS_2_ with a sub-nm focused helium ion beam to deterministically create defects, and obtain spectrally narrow emission lines that produce photons in the visible spectral range

## Introduction

Point defects are important for a variety of physical phenonema in semiconductors. For example, they provide a means to engineer the equilibrium free-carrier density, they can serve as quantum emitters capable of hosting discrete quantum spin systems that can be optically addressed^1^. A major challenge in several of these applications is that the defects need to be precisely positioned, which is particularly difficult for conventional three-dimensional semiconductors where defects are often buried deep within the bulk structure. One route to remedy this challenge is to reduce the physical dimensionality of the material. Over the last decade it became possible to fabricate and manipulate atomically thin two-dimensional (2D) materials that offer intriguing electronic and optoelectronic properties^2,3^. In particular, the hexagonal transition metal dichalcogenides (TMDCs), such as MoS_2_, MoSe_2_, WS_2_, and WSe_2_, are excellent candidates for photonic applications due to their exceptionally strong light-matter interaction resulting from weak non-local dielectric screening^4–6^. The strong Coulomb coupling manifests itself in an exciton dominated spectral response and very large exciton binding energies^4,5,7–9^.

Beyond the response of mobile excitons, also quantum dot-like emission from localized excitons was recently demonstrated in WSe_2_^10–14^, GaSe^15^, MoSe_2_^16^, and WS_2_^17^ and the nature of the potential, which localizes the excitons is still not fully understood. The origin of such quantum emitters was hitherto considered to be caused by uncontrolled strain potentials that locally reduce the band gap, thus, funneling the recombination of excitons via a discrete recombination center^17–20^. However, strain potentials are challenging to control, which limits the applicability of such quantum emitters for a prospective integration into quantum photonic circuits^20–25^. In particular, scalable quantum technologies require the development of controlled approaches for the direct planar integration of quantum emitters with atomic scale resolution.

In this work, we propose and investigate an alternative route to engineer defect emitters that are of atomic scale. Specifically, we demonstrate theoretically and experimentally that localized defects in two-dimensional layers create trapping potentials in which bound complexes are formed that emit in a range of about hundred meV below the free exciton line in MoS_2_. Our experimental approach to deterministically create such point defects involves site-selective helium ion exposure of small areas (100 × 100 nm) of atomically thin MoS_2_. The precision with which defects are localized is expected to be solely limited by the resolution of the helium ion beam. Compared to strain engineering, a controlled exposure with helium ions is superior due to the sub-nm beam size that is very well suited to structure 2D materials at the nanometer scale^26^. First attempts to generate optically active defects in TMDCs have been made by exposing the host crystal with *α*-particles^27^ and helium ions^28^. However, in these works a strong dynamical inhomogeneous linewidth broadening of ~40 meV was imposed on the emission due to the fluctuating environment. Here, we demonstrate that the encapsulation of helium irradiated MoS_2_ monolayers with hBN allows one to define defect emitters with very narrow linewidths. The combination of ion exposure and subsequent encapsulation results in a hitherto unobserved new class of spatially localized and spectrally narrow defect emission in MoS_2_ at very distinct energies on the order of ~100–220 meV below the neutral exciton, denoted by $\,{}^0X_{1s}^A$. Our spectroscopic observations are consistent with photon emission that stems from defect centers in MoS_2_, which serve as highly localized exciton emission centers. The interpretation is corroborrated by analyzing in detail the lineshape of the new emission peaks. This allows us to determine the interaction of the localized exciton with phonons, which indicates that the emitters are localized on a nanometer scale. Our study opens up new routes for inducing highly controllable defect emitters in two-dimensional materials to create arrays of coupled single-photon sources^1^, to study Anderson’s orthogonality catastrophe^29,30^, and to realize lattices of interacting excitons similarly to optical lattices for ultracold atomic gases^31^.

## Results

### Defect creation in a van der Waals heterostructure by helium ion irradiation

**Fig. 1:**
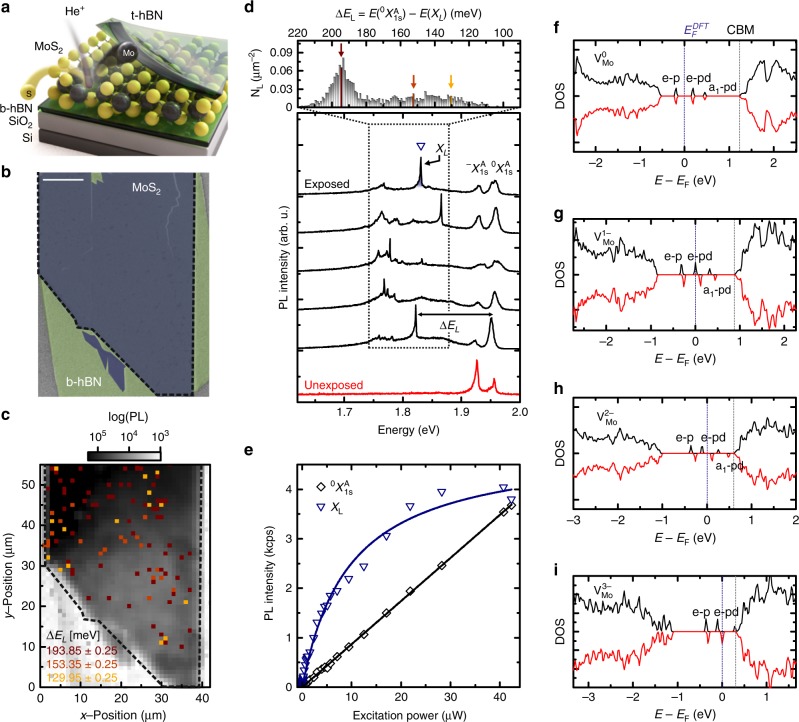
Deterministically induced defect emitters in atomically thin MoS_2_ realized by focused helium ions. **a** Schematic illustration of the exposed MoS_2_/hBN van der Waals heterostructure. **b** Helium ion microscopy image taken at a dose of *σ* = 2.2 ⋅ 10^12^ cm^−2^. The scale bar is 10 μm. **c** Spatially resolved and spectrally integrated photoluminescence mapping. The colored pixels depict the occurrence of emitters within 500 μeV wide energy bins (highlighted by colored arrows in **d**). **d** Bottom panel: typical low-temperature (10 K) *μ*-PL spectra of the exposed (black) and unexposed (red) hBN/MoS_2_/hBN heterostructure. The spectra of exposed MoS_2_ feature emission from mobile excitons $\,^0X_{1s}^A$ and trions $\,^ - X_{1s}^A$, as well as single emission lines *X*_*L*_ (open triangle) at lower energies. Top panel: a histogram of the emission energies detuned by Δ*E*_*L*_ = *E*($\,^0X_{1s}^A$) − *E*(*X*_*L*_) below $\,^0X_{1s}^A$. **e** Power dependence: $\,^0X_{1s}^A$ shows an expected linear power dependence, while the *X*_*L*_ emission saturates for higher excitation powers (data for the emitter highlighted by open triangle in **d**). **f**–**i** DFT calculated spin-up (black) and spin-down (red) density of states (DOS) of the neutral ${\mathrm{V}}_{Mo}^0$, single negatively ${\mathrm{V}}_{Mo}^{1 - }$, double negatively ${\mathrm{V}}_{Mo}^{2 - }$, and triple negatively charged ${\mathrm{V}}_{Mo}^{3 - }$ molybdenum vacancy. The DOS of ${\mathrm{V}}_{Mo}^0$ shows doublet *e*-*p*, *e*-*pd* and singlet *a*_1_-*pd* defect states inside the band gap. The *a*_1_-*p* singlet state is situated within the valence band. The more electrons are added to the vacancy the closer the defect states and the DFT computed Fermi level energy $E_F^{DFT}$ shift to the conduction band minimum (CBM) because of the on-site Coulomb repulsion

The van der Waals heterostrutures studied in this work are iteratively stacked onto Si substrates covered with 290 nm thermally grown SiO_2_. Onto this substrate we first transfer a multilayered hBN virtual substrate, and a single layer of MoS_2_. Typical hBN thicknesses vary between ~10–30 nm as confirmed by atomic force microscopy. The samples are transferred into a helium ion microscope, and are site-selectively exposed to helium ions at a constant dose of *σ* = 2.2 × 10^12^ cm^−2^, see Fig. 1a. Figure 1b shows a helium-ion microscope (HIM) image of the exposed van der Waals heterostructure. After the ion exposure, the entire MoS_2_ crystal is fully encapsulated by another multilayer hBN capping using a dry viscoelastic transfer method.

We perform low-temperature *μ*-photoluminescence (*μ*-PL) spectroscopy on the samples in a helium flow cryostat at a lattice temperature of 10 K. For excitation, we use a cw laser with a photon energy of 2.33 eV and a low excitation power (power density) of less than 10 μW (0.884 kW cm^−2^). Figure 1c shows the spatially resolved and spectrally integrated *μ*-PL response of a typical sample as a false color representation. Representative luminescence spectra from five randomly selected positions of the exposed sample that show defect emission are presented in the bottom of Fig. 1d and compared to a spectrum of a pristine (unexposed) sample. All spectra reveal spectrally narrow neutral exciton $\,^0X_{1s}^A$ (*FWHM* = (8.3 ± 2.5) meV) and charged exciton $\,^ - X_{1s}^A$ (*FWHM* = (9.4 ± 2.9) meV) emission whose inhomogeneous linewidth is reduced due to the encapsulation with hBN^6,32–34^. Remarkably, for the helium ion exposed samples in addition to the delocalized excitons, we observe spectrally sharper emission peaks *X*_*L*_ (*FWHM* ~ 0.5–6 meV) in a window of Δ*E*_*L*_ ~ 100–220 meV red-shifted from $\,^0X_{1s}^A$ (spectral range highlighted by a dotted box in Fig. 1d). These features are superimposed on the so-called L-peak that is observed in monolayer MoS_2_^4,5,35^ and has recently been attributed to the presence of sulphur vacancies^36,37^. The sharp spectral features are spatially localized and require helium ion exposure of MoS_2_ and the subsequent encapsulation with hBN. Spectrally sharp peaks do not occur in unexposed hBN/MoS_2_/hBN heterostacks (cf. bottom spectrum in Fig. 1d), neither in exposed MoS_2_ without hBN encapsulation nor in exposed hBN (See Supplementary Fig. 1).

To obtain further information on the origin of the sharp spectral emission, we statistically evaluate the energetic detuning Δ*E*_*L*_ = *E*($\,^0X_{1s}^A$) − *E*(*X*_*L*_) of each localized emission peak *E*(*X*_*L*_) with respect to *E*($\,^0X_{1s}^A$). Figure 1d, top, shows a histogram of the localized *X*_*L*_ emission energies as a function of detuning Δ*E*_*L*_ for ~3500 emitters for one representative sample (See Supplementary Fig. 5).

The histogram shows that the spectral emitters possess a continuum of energies in the range of Δ*E*_*L *_~ 100–220 meV below the neutral exciton. Intriguingly, we consistently observe that most of the helium ion induced emission peaks emit in a spectrally narrow (15) distribution at Δ*E*_*L*_ ~ 193 meV (also See Supplementary Figs 4 and 5).

We also show the spatial occurence of emitters in three energy bins (cf. arrows in Fig. 1d), overlayed in Fig. 1c. So far, single-photon emitters have been found at sample edges or folds, where the bandstructure is strongly influenced by the crystal boundary, coupling of atomic-orbitals by local symmetry breaking or strain^1^. By contrast, we find spectrally sharp emitters homogeneously distributed all over the 2D sample; this is a direct consequence of the helium ion exposure.

The sharp emission lines reveal a saturating behavior with a threshold power of *P*_*sat*_ ~ 10 μW (cf. Figure 1e), which is consistent with saturating a finite density of a single defect state as fitted by

1 $$I \propto \frac{P}{{P + P_{{\mathrm{sat}}}}} .$$

By contrast, the 2D neutral exciton $\,^0X_{1s}^A$ shows the expected linear dependence with the excitation power. MoS_2_ exhibits several defects, most prominently single sulfur vacancies and single molybdenum vacancies^38–40^. So far, sulphur vacancies have not been demonstrated to be optically active, because they are very likely to be passivated with oxygen^41^, although they are ubiquitous in exfoliated, unexposed MoS_2_^37^. Here, we concentrate on molybdenum vacancies V_*Mo*_, although we are aware that the discussed defect emitters might be of character that is more complex. Figure 1f–i show the ab-initio calculated density of states (DOS) for a neutral $V_{Mo}^0$, as well as a single, double and triple negatively charged molybdenum vacancy ($V_{Mo}^{1 - }$, $V_{Mo}^{2 - }$, and $V_{Mo}^{3 - }$). The vacancy states have a trigonal and mirror symmetry between the two layers of the sulphur and the molybdenum layer^40^. In turn, two singlet (*a*_1_) and two doublet (*e*) states are expected for all cases. The lowest *a*_1_-*p* [*e*-*p*] level stems from the six adjacent sulphur *p* orbitals, and are found inside [above] the valence bands. The *e*-*pd* and *a*_1_-*pd* states originate from the hybridization of the six sulphur *p* and six (second nearest neighbour) molybdenum *d* orbitals and are situated in the gap^40^. We find that all presented states are stable and relaxed within our DFT framework. Since Δ*E*_*L*_ is significantly smaller than the quasi particle band gap in MoS_2_, we assume that the Fermi energy is close to the conduction band minimum (CBM). We interpret the experimentally observed sharp emission lines as resulting from excitons that also involve particle-hole excitations from the defect state orbitals^42^.

Figure 1f–i demonstrates that for instance, for $V_{Mo}^{2 - }$ and $V_{Mo}^{3 - }$, the DFT computed Fermi energy $E_F^{DFT}$ is consistently close to the CBM. Taking the computed energy differences between each state and the CBM, our ab-initio calculation suggests that in this interpretation, the lowest unoccupied state in the $V_{Mo}^{3 - }$, the *e*-*pd* state, is likely to be involved in the photoluminescence signal *X*_*L*_ as in Fig. 1d (cf. Supplementary Figs 5 and 9). In particular, the computed energy difference Δ*E*^*DFT*^ = *E*(*CBM*)^*DFT*^ − *E(e-pd)*^*DFT*^ = 0.22 eV coincides with the highest experimental detuning Δ*E*_*L*_ in the top panel of Fig. 1d. However, the DFT calculation does not consider the binding energy of the exciton that is comprised of the different electronic orbitals with a complex distribution of weights and varying momentum dependencies^42^.

### Site-selective creation of optically active defects by local helium ion irradiation

**Fig. 2:**
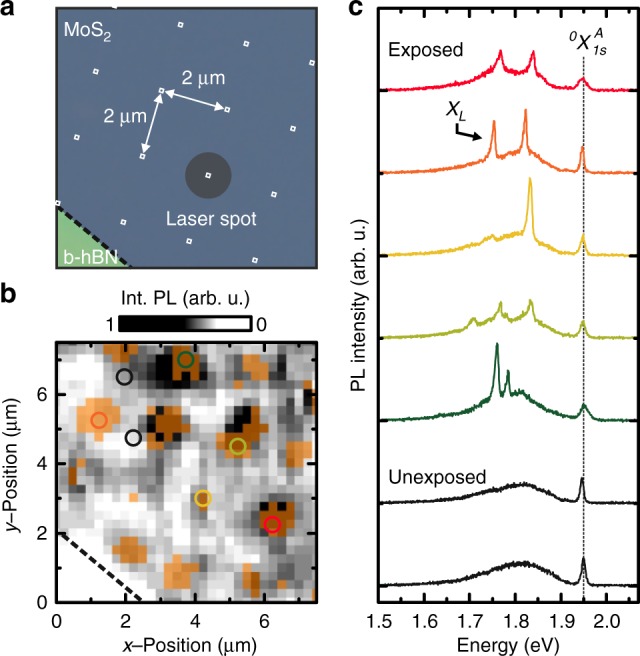
Site-selective generation of defect emission by helium ion exposure. **a** Optical micrograph of the monolayer MoS_2_/hBN van der Waals heterostructure prior to helium ion exposure and full encapsulation. A matrix of 100 × 100 nm fields is exposed with a pitch of 2 μm. A dose of *σ* = 2.2 × 10^12^ cm^−2^ is used. **b** Corresponding spatially resolved and spectrally integrated PL mapping (grayscale) at 10 K. Spectra are integrated for Δ*E* = (193 ± 5) meV. The spatial occurence of emitters in the range Δ*E*_*L*_ ~ 100–220 meV is overlayed in orange. **c** Selected spectra taken from **b** are shown. All spectra reveal emission from $\,^0X_{1s}^A$ while only irradiated areas reveal emission from localized states *X*_*L*_. The defect emission is absent in unexposed areas (black spectra and corresponding circles in **b**)

In order to demonstrate the site-selective nature of our approach, we write a matrix of 100 × 100 nm fields with a pitch of 2 μm onto monolayer MoS_2_ that is deposited on hBN. An optical micrograph of the flake prior to exposure is presented in Fig. 2a. Subsequent to exposure, the nano-structured MoS_2_ monolayer is fully encapsulated in hBN. We record a spatially resolved PL mapping of the treated areas with a small step size (250 nm). The corresponding spatially resolved and spectrally integrated PL mapping is presented in Fig. 2b (grayscale). Here, an integration range of Δ*E* = (193 ± 5) meV is chosen since, statistically (cf. Figure 1d), the localized emission is the strongest at these energies. Enhanced PL emission is clearly observed at the irradiated spots. Moreover, from the data, we extract and overlay the occurence of localized emission *X*_*L*_ with the PL mapping (highlighted in orange). We find that localized emission is activated at all positions that are exposed by the HIM. Conversely, sharp line emission is not observed from the unexposed regions. Corresponding spectra of five exposed positions are presented in Fig. 2c. Each spectrum exhibits a small number (1–3) of emission lines that are completely absent in unexposed areas (cf. black spectra in Fig. 2c). Importantly, the site-selectively created defect emitters exhibit emission at Δ*E* ~ 193 meV in agreement with the distribution obtained from statistics in Fig. 1d (also See Supplementary Fig. 5). Since no annealing step is needed for emitter creation, the writing accuracy is expected to be predominantly limited by the helium ion beam, and is, therefore expected to be on the nanometer scale. Near-field optical techniques and scanning tunneling microscopy can further be used to ultimately reveal the writing accuracy of atomistic defect states in the monolayer MoS_2_.

### Photoluminescence excitation spectroscopy and time stability of an individual single defect emitter

**Fig. 3:**
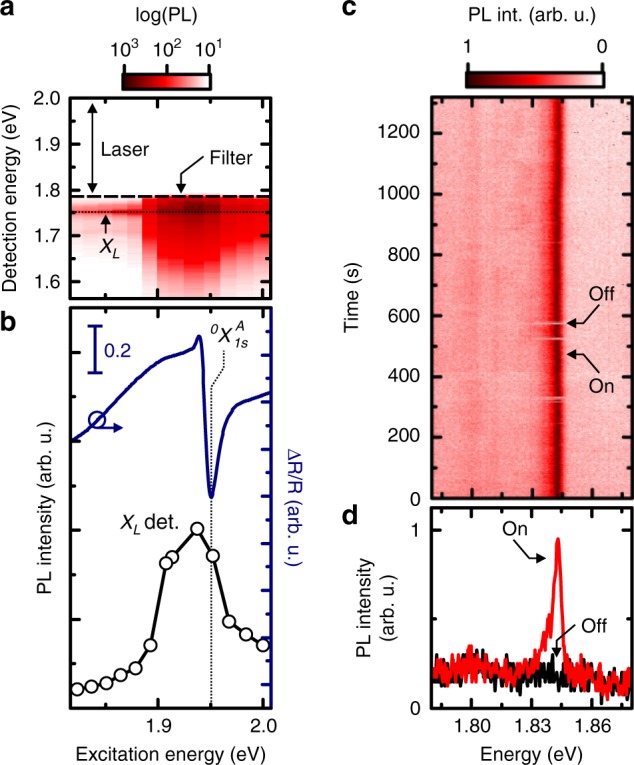
PL excitation spectroscopy and time stability of optically active defect emitters. **a** False color plot showing the localized emission *X*_*L*_ for the excitation being tuned across $\,^0X_{1s}^A$. **b** The differential reflectivity Δ*R*/*R* of the heterostructure reveals the $X_{1s}^A$ as highlighted by the dashed line. The reflectivity is compared to the PL intensity of the *X*_*L*_ as a function of laser excitation energy. **c** Time trace of the photoluminescence emission of a defect emitter recorded over long times. The integration time is set to 1 s while spectra are acquired every second. **d** Two exemplary spectra taken from **c** for the emitter being switched on and off

To further corroborate the above interpretation of optically active defects, we perform photoluminescence excitation (PLE) spectroscopy to probe the energetic structure of the involved states. Figure 3a presents a false color plot for a typical PLE scan of an emission line *X*_*L*_ as the excitation energy is tuned across the $\,^0X_{1s}^A$. The emission occurs at *E*(*X*_*L*_) = (1.76 ± 0.01) eV energetically well below the $\,^0X_{1s}^A$. When measuring differential reflectivity Δ*R*/*R* using a broadband supercontinuum source, we identify $\,^0X_{1s}^A$ at an energy of *E* ($^0X_{1s}^A$) = 1.95 eV (cf. Figure 3b). Upon resonant excitation of $^0X_{1s}^A$, the *X*_*L*_ emission is significantly increased. This is a consequence of the efficient creation of excitons, when the laser energy is on resonance with an exciton transition (See Supplementary Fig. 7 for excitation on $\,^0X_{2s}^A$ and $\,^0X_{1s}^B$). Most significantly, we can excite *X*_*L*_ states also below the free exciton transitions. This is consistent with recently predicted absorbance spectra of single vacancy defects in 2D materials^42^ and in contrast to strain induced quantum dot-like emitters^10–15,17–20^.

Another key property of photonic emitters is their spectral stability. A representative time trace of a defect emitter is presented in Fig. 3c (with a spectrum acquired every second). Examples of two spectra highlighted in Fig. 3c are plotted in Fig. 3d. They demonstrate that the emitter exhibits a blinking behavior. This is an observed feature of various types of few-level systems^43^. In our case, the blinking behavior is not surprising since the defects are embedded in a two-dimensional plane with large surface-to-volume ratio and charges in the environment that are very likely to randomly fluctuate in position and time.

### Defect-phonon interaction and the independent boson model

**Fig. 4:**
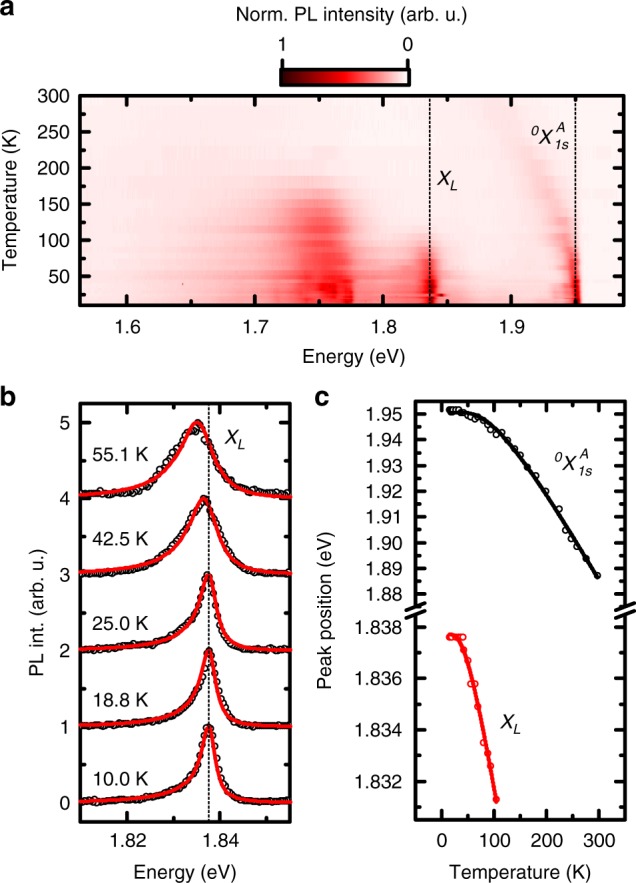
Temperature-dependent PL spectroscopy of a single emission line *X*_*L*_ and independent boson model. **a** False color plot of the temperature-dependent evolution of PL from localized and delocalized excitons. The emission energies of neutral exciton $\,^0X_{1s}^A$ and one defect emitter *X*_*L*_ at 10 K are highlighted with a dashed line, respectively. **b** Temperature-dependent spectra of *X*_*L*_ fitted with an independent boson model. Best agreement is found for *a*_*B*_ = 2 nm. **c** Temperature-dependent peak position of $\,^0X_{1s}^A$ and *X*_*L*_. Data are fitted with Eq. (2)

Measuring the temperature dependence of the *μ*-PL spectra allows us to determine the interactions between the involved electron orbitals and phonons. We gradually change the lattice temperature from 10 K to 300 K while recording *μ*-PL spectra (cf. Figure 4a). The spectra reveal emission from the defect emitters that are strongly red-shifted and simultaneously broadened at elevated temperatures (similar as for the $\,^0X_{1s}^A$). We analyze the evolution of the emitter *X*_*L*_ in detail by utilizing the independent boson model that has been successfully applied to the lineshape analysis of quantum dot states^44–46^ and defect-bound excitons^47^. Figure 4b highlights the spectra of *X*_*L*_ for selected temperatures with lineshapes fitted by this model (red lines). In particular, the lineshape of each emitter is found to exhibit a very pronounced acoustic phonon sideband (~10 meV) at the low energy side. Most likely, this low energy tail results from interaction with a specific bandwidth of acoustic phonons since the defects are highly localized in real space^42^. Our model particularly accounts for the coupling with LA/TA phonon branches, while coupling with the ZA branch is found to be negligible. For low temperatures, we find the best agreement of the calculated lineshape with our data for an effective Bohr radius of *a*_*B*_ = 2 nm. Taking into account a theoretical phonon lifetime of $\frac{1}{{\gamma _{ph}}}\sim 40\,{\mathrm{ps}}$ ^48^, we obtain an excitonic radiative lifetime (linewidth) of ~1 ps (~0.5 meV). The lineshape in Fig. 4b is most asymmetric at low temperatures where phonon emission is more likely to happen than phonon absorption while for higher temperatures, the lineshape becomes more symmetric. Moreover, the PL emission reduces in intensity at elevated temperatures with the defect emitters typically exhibiting activation energies that are far below *k*_*B*_*T* (See Supplementary Fig. 10).

The temperature-dependent peak positions in Fig. 4a manifest in a polaron shift for all spectral features (cf. Figure 4c), as phenomenologically described by^49^

2 $$E_G(T) = E_G(0) - S\langle \hbar \omega \rangle \left[ {{\mathrm{coth}}\frac{{\langle \hbar \omega \rangle }}{{2k_BT}} - 1} \right],$$

with the emission energy *E*_*G*_(0) at 0 K, the Huang–Rhys factor *S*, the average phonon energy 〈ℏ*ω*〉, and the Boltzmann constant *k*_*B*_. Fitting *X*_*L*_ in Fig. 4c reveals average phonon energies of 〈ℏ*ω*〉 = (12.69 ± 1.17) meV and an electron–phonon coupling of *S* = 0.791 ± 0.071 (for $\,^0X_{1s}^A$: 〈*ℏω*〉 = (25.11 ± 1.02) meV and *S* = 2.135 ± 0.061). The experimentally observed value of *S*_*exp*_ = 0.791 is in very good agreement with *S*_*theory*_ = 0.75 as obtained from the independent boson model (See Supplementary Fig. 12). From our model, we can also distinguish between the fraction of photons emitted by the zero phonon line (ZPL) and the phonon sideband (PSB). Taking the relative spectral weight of ZPL and PSB, we find a Debye–Waller factor of ~0.2 at 10 K, comparable to other few-level systems^50^.

## Discussion

To start the discussion, we point out that the emission peaks occur homogeneously across the whole basal plane of the exposed MoS_2_ encapsulated into hBN (cf. Figure 1c). The sharp peaks neither occur in exposed hBN without MoS_2_ nor in unexposed hBN/MoS_2_/hBN (See Supplementary Fig. 1). This phenomenology suggests that the optically active defects can probably not be explained by sulphur vacancies, because the latter are ubiquitous in exfoliated, unexposed MoS_2_^37^, although they are very likely to be produced under helium ion exposure as well^28,38,40^. However, sulphur vacancies are easily passivated by oxygen^41^, which especially applies to the discussed nano-fabricated TMDCs under ambient conditions. Figure 2b, c support this interpretation, since the emission *X*_*L*_ only appears at the exposed areas, while the unexposed areas show the broad L-peak attributed to the presence of sulphur vacancies^36,37^. Equally, quantum emitters in hBN can be excluded for our sample (See Supplementary Figs 1 and 8)^51^. Intriguingly, the beam energy of the HIM (30 keV) and also the excess energy of the secondary electrons (<10 eV) produced in the exposed materials are sufficient to form defects with a large formation energy, such as molybdenum vacancies^40,52^, that are rare in pristine samples^37,39^. This argument could explain why we observe optically active defect sites only after the helium ion exposure of the MoS_2_ monolayers.

In our understanding, the influence of hBN is two-fold. On the one hand, the emission peaks *X*_*L*_ have the lowest emission energy of Δ*E*_*L*_ of ~193 meV below $\,^0X_{1s}^A$. Encapsulated in hBN, the excitonic emission energy is renormalized due to the dielectric environment^6^. On the other hand, the emission linewidth is strongly reduced for both *X*_*L*_ and $\,^0X_{1s}^A$^6,32–34^. The combination of the hBN-effects explain the sharp spectral features, which are spread across the broad energy window Δ*E*_*L*_ ~ 100–220 meV in Fig. 1d. However, most of the defects emit at an energy detuning of ~193 meV, which resembles the spectrally broad PL emission of helium ion exposed MoS_2_ on SiO_2_/Si substrate^28^ and on hBN (See Supplementary Fig. 2). This observation is also in good agreement with the emission properties of site-selectively activated defects in Fig. 2. From our consistent observations, we draw the conclusion that most of the emission indeed originates from the same helium ion induced defect that is deterministically created at designated positions in the MoS_2_ monolayer. The defect emission at smaller detunings Δ*E*_*L*_ < 180 meV is most likely caused by dielectric effects, adsorbates or simply by another type of defect.

For all possibly created defects, the emission at Δ*E*_*L*_ of ~193 meV must resemble the lowest unoccupied, optically active state. This interpretation is consistent with the saturating behavior as in Fig. 1e, and it also explains the blinking behavior in Fig. 3c, d. Furthermore, the PLE measurements in Fig. 3a are also consistent with this interpretation, since the expected joint density of states of localized electron orbitals at the defect and its environment sum up to a finite, continuous absorbance from the lowest unoccupied state (i.e., at Δ*E*_*L*_ of ~193 meV) up to $\,^0X_{1s}^A$^42^. The excitation laser creates electrons that can occupy the defect states followed by optical recombination with a valence band hole and possible further local defect orbitals^42^.

We would like to note that there are few possible defects in MoS_2_, from the already mentioned sulphur and molybdenum vacancies to interstitials and defect complexes involving more than one atom.^38–40^. For the sake of clarity, we concentrate on *V*_*Mo*_. Our DFT calculation in Fig. 1 neglects the momentum dependence of the involved electron states and variable binding energies per orbital^42^. In our experiment, the influence of ambient gases might have further effects^28^. Taking the argument of refs. ^40,42^, the complex admixture of *p*- and *d*-orbitals in the molybdenum defect (cf. Figure 1f–i) give rise to a continuous absorbance below $\,^0X_{1s}^A$, as recently discussed for single chalcogen vacancies^40,42^.

Figure 4b demonstrates that the effective exciton–phonon interaction can be described by the independent boson model, which suggests that the effective interaction length is localized within only ~2 nm. The independent boson model further suggests an excitonic lifetime of only 1 ps (See Supplementary Fig. 12). This theoretical value is a lower bound for the actual lifetime because we omit other sources of broadening mechanisms. Experimentally, we determine the upper bound of the radiative lifetime to be <150 ps (limited by the instrument response function), which renders second order time-correlation measurements of g^(2)^ challenging. This experimental observation is in stark contrast to strain induced quantum dot-like emitters in TMDCs with nanosecond lifetimes based on an effective type-II band alignment^10–14^. Different excitation conditions in gated devices that could suppress the L-peak emission may enable to access the photon statistics and unambiguously proof the quantum nature of the defect emission.

In conclusion, we have demonstrated a method to deterministically and site-selectively generate optically active defects in MoS_2_. The superior optical quality resulting from hBN encapsulation reveals spectrally narrow emission at very distinct detunings with respect to the neutral exciton. The emission lines show a saturating power dependence and a blinking behavior. On a broader perspective, a controlled production of optically active defects in a periodic pattern using helium ion beam exposure may allow the exploration of exotic many-body physics in lattice systems, including the demonstration of coupled photon sources^1^, the Anderson orthogonality catastrophe^29,30^, as well as the Mott transition between a superfluid and an insulator^31^ in the presence of dissipation. The site-selective approach for writing optically active defects with high accuracy and ease at designated positions is expected to be highly beneficial for integration into photonic circuits. Moreover, the inherent proximity of the engineered defect states to surfaces opens exciting ways for possibly harnessing them as local nanometer scale sensors, investigating nuclear spin physics with wave functions only sampling a fraction of atoms or studying screening physics in the ultimate limit of a localized electronic defect state.

## Methods

### Sample structure

We employed the viscoelastic transfer method to transfer MoS_2_ monolayer crystals and hBN multilayers onto 290 nm SiO_2_ substrates. The MoS_2_ crystals used for exfoliation were purchased from SPI Supplies while high-quality hBN bulk crystals were provided by Takashi Taniguchi and Kenji Watanabe from NIMS, Japan.

### Helium ion microscopy

We used MoS_2_ monolayers transfered onto hBN/SiO_2_/Si substrates for He^+^ irradiation. For the data shown in Fig. 1, a beam current *I* ~ 1 pA and a beam energy of 30 kV are used. Large areas are exposed with a beam spacing of 5 nm. The dwell time was adjusted such that a dose of ~10^12^ He^+^ cm^−2^ is obtained, which is optimized for a high density of defect emitters (See Supplementary Fig. 6).

For the data shown in Fig. 2, a low beam current of *I* ~ 20 fA is used in order to obtain a low helium ion dose of ~10^12^ He^+^ cm^−2^ by accordingly adjusting the dwell time. Small areas of 100 × 100 nm are exposed. A beam spacing of 0.5 nm is used.

### Optical spectroscopy

Confocal optical spectroscopy was performed in a helium flow cryostat with the sample kept at a lattice temperature of ~10 K. For cw *μ*-PL experiments in Fig. 1, we used a doubled Nd:YAG laser with an excitation energy of 2.33 eV and typical excitation powers of less than 10 μW, which results in an excitation power density of 0.884 kW cm^−2^ at a laser spot diameter of 1.2 μm.

The *μ*-PL data in Fig. 2a are recorded with an excitation energy of 2.54 eV and an excitation power (density) of 900 nW (0.058 kW cm^−2^) with a laser spot diameter of 0.7 μm.

For measuring differential reflectivity spectra a supercontinuum white light source was used that was focused with an optical microscope onto the sample kept at a lattice temperature of ~10 K with a spot size of ~1–2 μm.

For PLE experiments, we used an optical parametric oscillator (OPO) pumped with a Ti:Sa at 820 nm with a repetition rate of 80 MHz and a 150 fs pulse width. For our measurements, we used the signal output of the OPO, which was incrementally tuned in steps of ~25 meV from 1.8–2.45 eV. The excitation power was kept at 20 μW for all excitation energies. The excitation laser pulse was filtered with a sharp edge filter before being dispersed on a grating and detected with a charge-coupled device (CCD).

### DFT calculations

The calculations were performed using density functional theory (DFT) as implemented in the Vienna ab-initio simulation package (VASP)^53,54^. The projected augmented wave method has been used^55,56^. The atomic and electronic structures were determined using the PBE functional. A plane wave basis with an energy cutoff of E_cut_ = 500 eV and a (6 × 6 × 1) Monkhorst-Pack **k**-point sampling has been used. The TMD layer has been modeled using a (9 × 9) supercell containing 242 atoms.

## Supplementary information

Supplementary Information

## Data Availability

The data that support the findings of this study are available from the corresponding author on reasonable request.

## References

[1] Aharonovich I, Englund D, Toth M (2016). Solid-state single-photon emitters. Nat. Photonics.

[2] Ajayan P, Kim P, Banerjee K (2016). Two-dimensional van der waals materials. Physics Today.

[3] Roldán R (2017). Theory of 2d crystals: graphene and beyond. Chem. Soc. Rev..

[4] Mak KF, Lee C, Hone J, Shan J, Heinz TF (2010). Atomically thin MoS_2_: a new direct-gap semiconductor. Phys. Rev. Lett..

[5] Splendiani A (2010). Emerging photoluminescence in monolayer MoS_2_. Nano Lett..

[6] Florian M (2018). The dielectric impact of layer distances on exciton and trion binding energies in van der waals heterostructures. Nano Lett..

[7] Ugeda MM (2014). Giant bandgap renormalization and excitonic effects in a monolayer transition metal dichalcogenide semiconductor. Nat. Mater..

[8] Chernikov, A. et al. Exciton binding energy and nonhydrogenic Rydberg series in monolayer WS_2_. *Phys. Rev. Lett.* **113**, 076802 (2014). 10.1103/PhysRevLett.113.076802 25170725

[9] He K (2014). Tightly bound excitons in monolayer WSe_2_. Phys. Rev. Lett..

[10] Tonndorf P (2015). Single-photon emission from localized excitons in an atomically thin semiconductor. Optica.

[11] Srivastava A (2015). Optically active quantum dots in monolayer WSe_2_. Nat. Nanotechnol..

[12] He Y-M (2015). Single quantum emitters in monolayer semiconductors. Nat. Nanotechnol..

[13] Koperski M (2015). Single photon emitters in exfoliated WSe_2_ structures. Nat. Nanotechnol..

[14] Chakraborty C, Kinnischtzke L, Goodfellow KM, Beams R, Vamivakas AN (2015). Voltage-controlled quantum light from an atomically thin semiconductor. Nat. Nanotechnol..

[15] Tonndorf P (2017). Single-photon emitters in GaSe. 2D Mater..

[16] Branny A (2016). Discrete quantum dot like emitters in monolayer MoSe_2_: spatial mapping, magneto-optics, and charge tuning. Appl. Phys. Lett..

[17] Palacios-Berraquero C (2017). Large-scale quantum-emitter arrays in atomically thin semiconductors. Nat. Commun..

[18] Kern J (2016). Nanoscale positioning of single-photon emitters in atomically thin WSe_2_. Adv. Mater..

[19] Branny A, Kumar S, Proux R, Gerardot BD (2017). Deterministic strain-induced arrays of quantum emitters in a two-dimensional semiconductor. Nat. Commun..

[20] Blauth M (2018). Coupling single photons from discrete quantum emitters in WSe_2_ to lithographically defined plasmonic slot waveguides. Nano Lett..

[21] Laucht, A. et al. A waveguide-coupled on-chip single-photon source. *Phys. Rev. X* **2**, 011014 (2012).

[22] Reithmaier G (2015). On-chip generation, routing, and detection of resonance fluorescence. Nano Lett..

[23] Goodfellow KM, Beams R, Chakraborty C, Novotny L, Vamivakas AN (2014). Integrated nanophotonics based on nanowire plasmons and atomically thin material. Optica.

[24] Goodfellow KM, Chakraborty C, Beams R, Novotny L, Vamivakas AN (2015). Direct on-chip optical plasmon detection with an atomically thin semiconductor. Nano Lett..

[25] Blauth M, Harms J, Prechtl M, Finley JJ, Kaniber M (2017). Enhanced optical activity of atomically thin MoSe_2_ proximal to nanoscale plasmonic slot-waveguides. 2D Mater..

[26] Fox DS (2015). Nanopatterning and electrical tuning of MoS_2_ layers with a subnanometer helium ion beam. Nano Lett..

[27] Tongay, S. et al. Defects activated photoluminescence in two-dimensional semiconductors: interplay between bound, charged and free excitons. *Sci. Rep.* **3**, 2657 (2013). 10.1038/srep02657 PMC3772378 24029823

[28] Klein J (2017). Robust valley polarization of helium ion modified atomically thin MoS_2_. 2D Mater..

[29] Anderson PW (1967). Infrared catastrophe in fermi gases with local scattering potentials. Phys. Rev. Lett..

[30] Schmidt R (2018). Universal many-body response of heavy impurities coupled to a fermi sea: a review of recent progress. Rep. Prog. Phys..

[31] Bloch I, Dalibard J, Zwerger W (2008). Many-body physics with ultracold gases. Rev. Mod. Phys..

[32] Wierzbowski, J. et al. Direct exciton emission from atomically thin transition metal dichalcogenide heterostructures near the lifetime limit. *Sci. Rep.* **7**, 12383 (2017). 10.1038/s41598-017-09739-4 PMC5620059 28959034

[33] Ajayi OA (2017). Approaching the intrinsic photoluminescence linewidth in transition metal dichalcogenide monolayers. 2D Mater..

[34] Cadiz, F. et al. Excitonic linewidth approaching the homogeneous limit in MoS_2_-based van der Waals heterostructures. *Phys. Rev.* **7**, 2 (2017).

[35] Korn T, Heydrich S, Hirmer M, Schmutzler J, Schüller C (2011). Low-temperature photocarrier dynamics in monolayer MoS_2_. Appl. Phys. Lett..

[36] Carozo V (2017). Optical identification of sulfur vacancies: bound excitons at the edges of monolayer tungsten disulfide. Sci. Adv..

[37] Klein, J. et al. Impact of intrinsic and extrinsic imperfections on the electronic structure of monolayer MoS_2_. *arXiv:1905*.*01242* (2019).

[38] Komsa, H.-P. et al. Two-dimensional transition metal dichalcogenides under electron irradiation: Defect production and doping. *Phys. Rev. Lett.* **109**, 035503 (2012). 10.1103/PhysRevLett.109.035503 22861869

[39] Hong, J. et al. Exploring atomic defects in molybdenum disulphide monolayers. *Nat. Commun.* **6**, 6293 (2015). 10.1038/ncomms7293 PMC4346634 25695374

[40] Noh J-Y, Kim H, Kim Y-S (2014). Stability and electronic structures of native defects in single-layer mos_2_. Phys. Rev. B.

[41] Barja, S. et al. Identifying substitutional oxygen as a prolific point defect in monolayer transition metal dichalcogenides with experiment and theory. *arXiv:1810*.*03364* (2018). 10.1038/s41467-019-11342-2 PMC6662818 31358753

[42] Refaely-Abramson, S., Qiu, D. Y., Louie, S. G. & Neaton, J. B. Defect-induced modification of low-lying excitons and valley selectivity in monolayer transition metal dichalcogenides. *Phys. Rev. Lett.* **121**, 167402 (2018). 10.1103/PhysRevLett.121.167402 30387666

[43] Efros AL, Nesbitt DJ (2016). Origin and control of blinking in quantum dots. Nat. Nanotechnol..

[44] Zimmermann, R. & Runge, E. Dephasing in quantum dots via electron-phonon interaction. *Proc*. *26th ICPS, Edinburgh* (2002).

[45] Wilson-Rae I, Imamoğlu A (2002). Quantum dot cavity-QED in the presence of strong electron-phonon interactions. Phys. Rev. B.

[46] Krummheuer, B., Axt, V. M. & Kuhn, T. Theory of pure dephasing and the resulting absorption line shape in semiconductor quantum dots. *Phys. Rev.* **65**, 195313 (2012).

[47] Duke CB, Mahan GD (1965). Phonon-broadened impurity spectra. i. density of states. Phys. Rev..

[48] Gu X, Li B, Yang R (2016). Layer thickness-dependent phonon properties and thermal conductivity of MoS_2_. J. Appl. Phys..

[49] O’Donnell KP, Chen X (1991). Temperature dependence of semiconductor band gaps. Appl. Phys. Lett..

[50] Atatüre M, Englund D, Vamivakas N, Lee S-Y, Wrachtrup J (2018). Material platforms for spin-based photonic quantum technologies. Nat. Rev. Mater..

[51] Tran TT, Bray K, Ford MJ, Toth M, Aharonovich I (2015). Quantum emission from hexagonal boron nitride monolayers. Nat. Nanotechnol..

[52] Ohya K, Yamanaka T, Inai K, Ishitani T (2009). Comparison of secondary electron emission in helium ion microscope with gallium ion and electron microscopes. Nucl. Instrum. Methods Phys. Res. Sect. B.

[53] Kresse G, Furthmüller J (1996). Efficiency of ab-initio total energy calculations for metals and semiconductors using a plane-wave basis set. Comput. Mater. Sci..

[54] Kresse G, Furthmüller J (1996). Efficient iterative schemes forab initiototal-energy calculations using a plane-wave basis set. Phys. Rev. B.

[55] Kresse G, Joubert D (1999). From ultrasoft pseudopotentials to the projector augmented-wave method. Phys. Rev. B.

[56] Blöchl PE (1994). Projector augmented-wave method. Phys. Rev. B.

