# Supplementary material for: Site-selectively generated photon emitters in monolayer MoS2 via local helium ion irradiation
Source: Nat Commun. 2019 Jun 21;10:2755. doi: 10.1038/s41467-019-10632-z (PMC6588625; doi:10.1038/s41467-019-10632-z)
Supplement: Supplementary file 1 — Supplementary Information [file 41467_2019_10632_MOESM1_ESM.pdf]

Supplementary Information - Site-selectively generated photon emitters in monolayer MoS_2 via local helium ion irradiation

Klein et al.

Supplementary Note 1: Optical spectroscopy of He-ion bombarded hBN

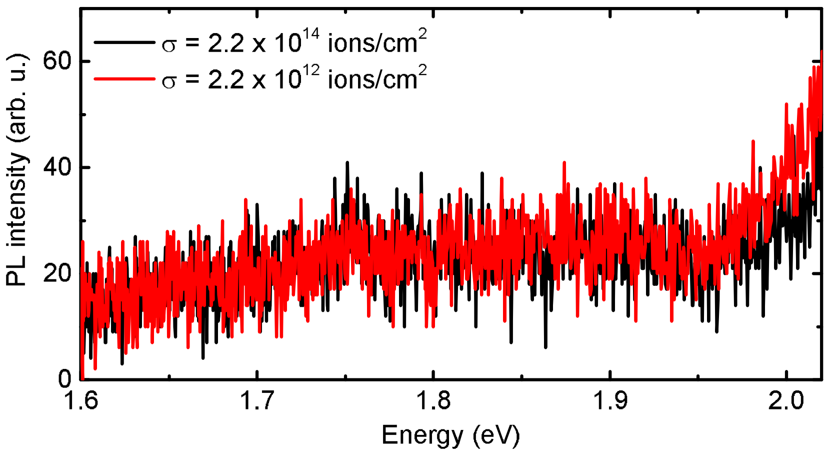

Supplementary Figure 1. **Photoluminescence of He-ion bombarded hBN.** Two exemplary spectra taken from a spatially resolved low-temperature (10 K) μ-PL mapping on He-ion bombarded hBN with doses of $\sigma = 2.2 \cdot 10^{12}$ ions cm^{-2} (red) and $\sigma = 2.2 \cdot 10^{14}$ ions cm^{-2} (black).

Recently, quantum emission from optically active defects in hBN crystals has been demonstrated. [1] In order to rule out any defect emission contribution to the defect emission from the hBN crystals, we irradiate large areas (>400 µm^2) of exfoliated and deterministically transferred hBN flakes on the same SiO_2/Si substrates that are used for all experiments of this work. Two spectra taken from low temperature (10 K) spatially resolved μ-PL mappings recorded for He-ion doses of $\sigma = 2.2 \cdot 10^{12}$ ions cm^{-2} (red) and $\sigma = 2.2 \cdot 10^{14}$ ions cm^{-2} (black) are shown in Supplementary Figure 1. Besides background noise from the CCD chip used for integration in our experiments, our data shows no indication of quantum emission from hBN induced by the He-ions in all bombarded areas.

Supplementary Note 2: Dose dependent spectra of non-encapsulated MoS_2

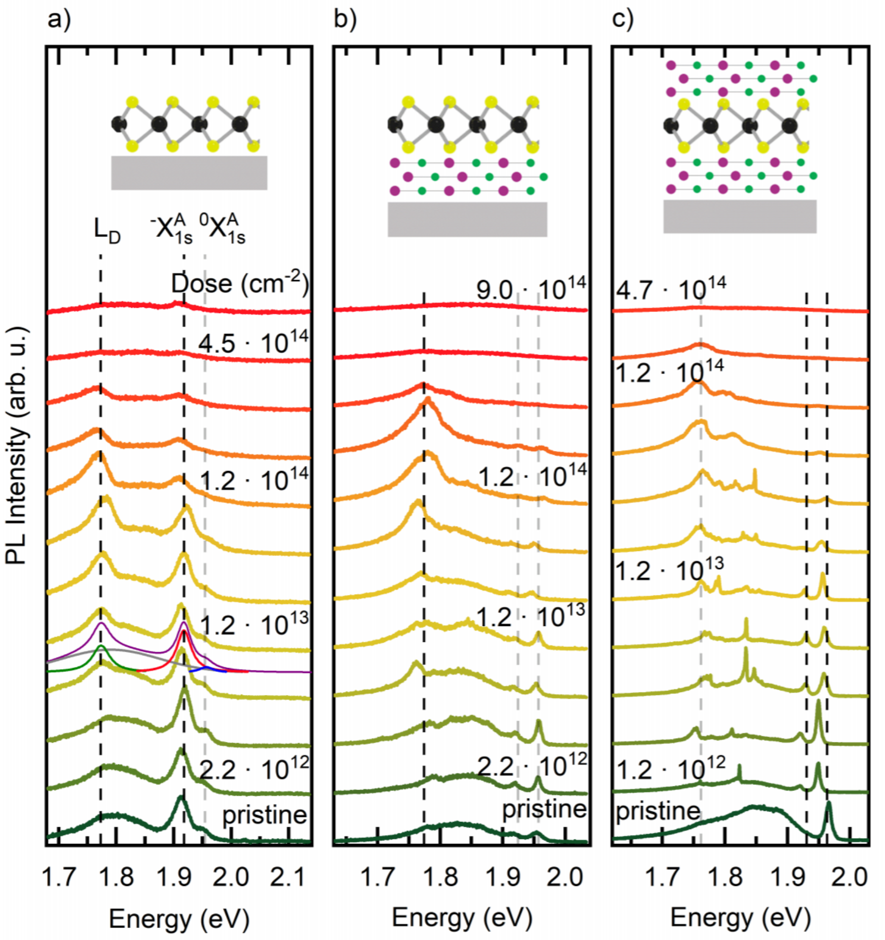

Supplementary Figure 2. **He-ion dose dependent photoluminescence spectra of MoS_2 in different dielectric environments.** **a**, Dose dependent photoluminescence of MoS_2 on SiO_2/Si. The spectrum reveals emission from the neutral exciton ${}^0X^A_{1s}$, and charged exciton ${}^-X^A_{1s}$, and from the L-peak and furthermore dose dependent emission from the L_D peak. (Data are adapted from Ref. [2]) **b**, Dose dependent photoluminescence of MoS_2 on hBN reveals spectrally more narrow free exciton emission and similar to **a** emission from the L-peak and dose dependent defect emission from the L_D-peak **c**, Dose dependent photoluminescence of a hBN/MoS_2/hBN van der Waals heterostructure. Besides spectrally narrow free exciton emission the spectrum also reveals spectrally sharp emission.

Since the photo-physical properties of single-layer MoS_2 strongly depend on the dielectric environment, especially on the encapsulation and passivation of the surface of the crystal,[3–6] we spectroscopically investigate the He-ion dose dependence of single-layer MoS_2 in differ-

ent dielectric environments. Supplementary Figure 2 shows typical low-temperature (10 K) μ-PL spectra of single-layer MoS_2 on SiO_2/Si, MoS_2/hBN and fully hBN encapsulated MoS_2. The He-ion dose is varied between $\sigma \sim 10^{12}$ ions cm^{-2} and $\sigma \sim 10^{15}$ ions cm^{-2}. All spectra reveal emission from the neutral and charged exciton with much narrower linewidths on hBN substrates due to a reduced inhomogeneous linewidth. [4–7] Moreover, all spectra exhibit typically observed broad low-energy emission from the L-peak in addition to a superimposed emission from the L_D peak [2] that increases with increased σ. This emission is detuned by $\Delta E \sim 190$ meV from the neutral exciton and spectrally narrows for fully hBN encapsulated MoS_2. Remarkably, only sandwiched MoS_2 reveals very sharp emission. The overall PL emission in all three sample geometries quenches for $\sigma > 10^{14}$ ions cm^{-2} which is likely due to high defect densities.

Supplementary Note 3: Spatially localized defect emission in vdW heterostructures

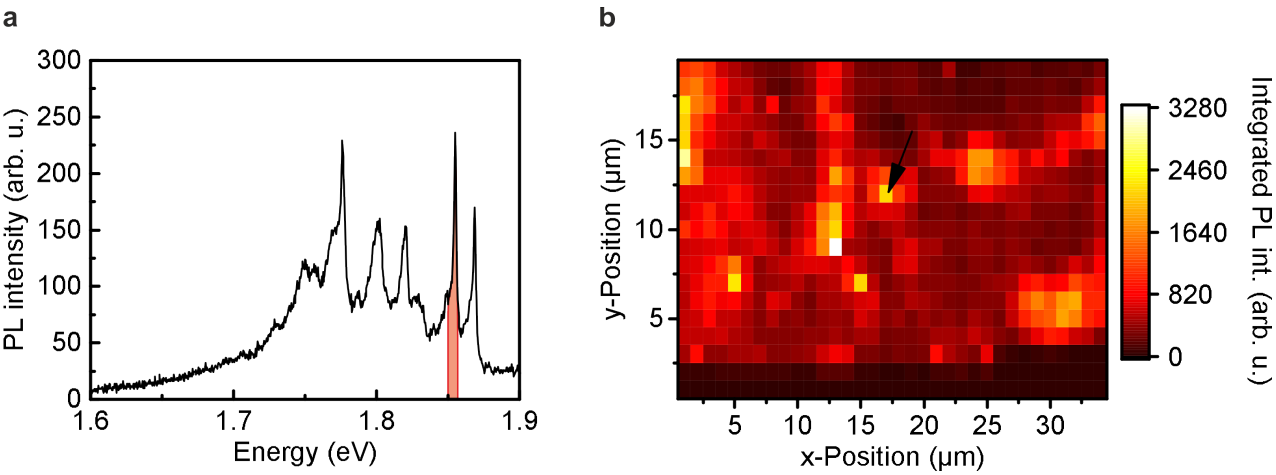

Supplementary Figure 3. Spatially localized photoluminescence in hBN/MoS_2/hBN. **a**, Typical photoluminescence spectrum that features emission from different single defect emitters. **b**, Corresponding spatially integrated PL mapping of the red coloured line in **a**. The spatial position from which the spectrum is taken from is highlighted by the black arrow. The photoluminescence is spatially localized within the focal spot diameter of the confocal microscope.

To spatially correlate the spectrally sharp emission, we perform spatially resolved μ-PL mappings. Supplementary Figure 3a shows a typical photoluminescence spectrum at 10 K. The spectrum reveals various single defect emitters. The corresponding spatially integrated PL mapping for the red coloured emitter is shown in Supplementary Figure 3b. The arrow highlights the spatial position from which the spectrum is taken from. The emission is spatially localized with a spatial extent that is limited by the focal spot diameter ($\sim 1.2\,\mu\text{m}$) of our confocal microscope.

Supplementary Note 4: Averaged photoluminescence spectrum of He-ion bombarded hBN/MoS_2/hBN

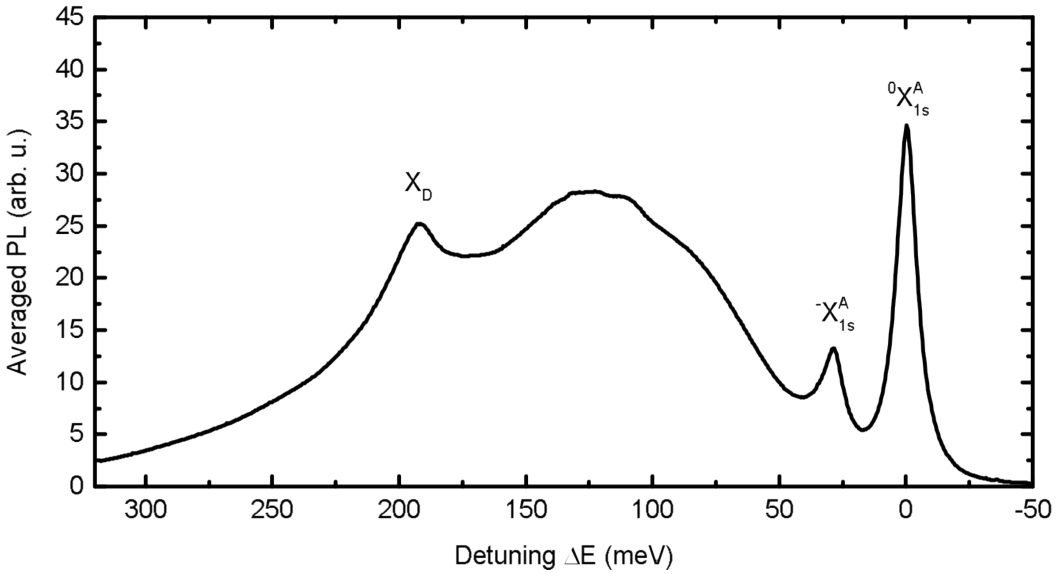

Supplementary Figure 4. **Averaged photoluminescence spectrum of defective, and hBN encapsulated MoS_2.** The spatially averaged photoluminescence spectrum shows emission from neutral and charged exciton and also significant contribution from the L-peak and from the defect X_D-peak. The neutral exciton is set to zero detuning ΔE.

Supplementary Figure 4a shows an averaged PL spectrum with the neutral exciton set to zero detuning ΔE. Here, we average over all spectra that reveal emission from the neutral exciton and emission from localized emitters. In each spectrum the emission energy of the neutral exciton is used as a reference in order to properly sum up all spectra. The averaged spectrum shows emission from the neutral and charged exciton, a significant contribution from the L-peak at lower energies and also a significant contribution from the most prominent defect peak X_D at $\Delta E \sim 190\,\text{meV}$. The spectrum has similarities with a photoluminescence spectrum of He-ion bombarded MoS_2 on SiO_2 as reported recently [2] and also shown in Supplementary Figure 2a.

Supplementary Note 5: Spectral occurence of single defect emission

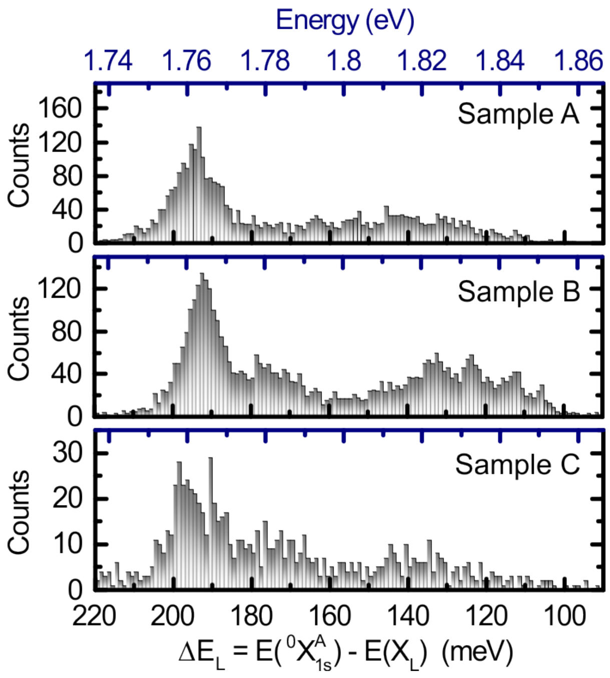

Supplementary Figure 5. **Photoluminescence of He-ion bombarded hBN.** Histogram of the energy detuning ΔE_L of single defect emission X_L for three samples. Data are obtained from low-temperature (10 K) spatially resolved μ-PL spectroscopy.

To verify the reproducibility of spectral occurence of single defect emission, we repeated experiments on two additional helium ion bombarded heterostacks. To this end, perform low-temperature (10 K) spatially resolved μ-PL spectroscopy on three nominally identical heterostacks. Emission statistics for all three measured samples are presented in Supplementary Figure 5. All histograms qualitatively reveal the same distribution, showing clustering at $\Delta E \sim 190\,\mathrm{meV}$ and a broader emission band for $< \Delta E \sim 190\,\mathrm{meV}$.

Supplementary Note 6: Dose dependent occurence of single defect emitters

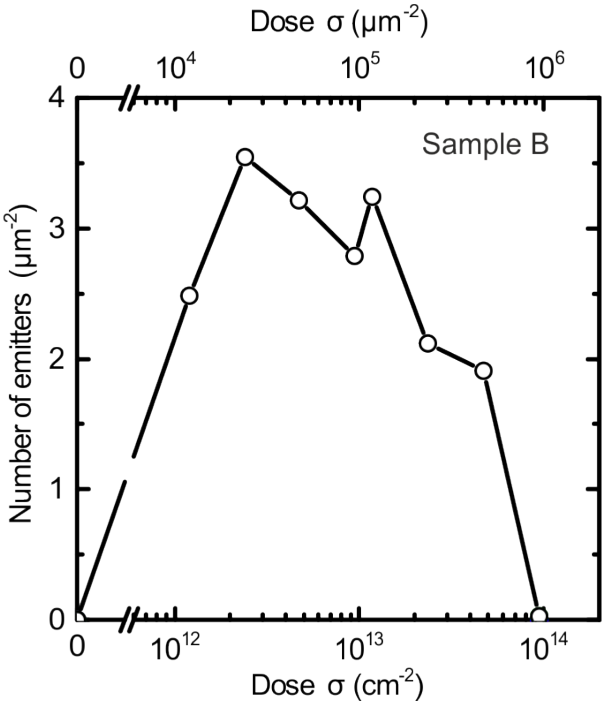

Supplementary Figure 6. **Dose dependent occurence of single defect emission.** The dose dependent number of emitters shows a maximum at a dose of $\sigma \sim 2 \cdot 10^{12}\text{cm}^{-2}$ with a steep decrease for $\sigma > 7 \cdot 10^{13}\text{cm}^{-2}$.

Instead of creating defects with a fixed helium ion dose, we continuously vary the ion dose over two orders of magnitude from $\sigma = 2.2 \cdot 10^{12}\text{cm}^{-2}$ to $\sigma = 1.4 \cdot 10^{14}\text{cm}^{-2}$ by adjusting the dwell time accordingly. Here, large fields of $4 \times 8\,\mu\text{m}$ are exposed for each dose in order to create reliable statistics. The creation efficiency of single defect emitters has a maximum at $\sim 2 \cdot 10^{12}\text{cm}^{-2}$ corresponding to ~ 3.5 emitters μm^{-2}.

Supplementary Note 7: Photoluminescence excitation spectroscopy

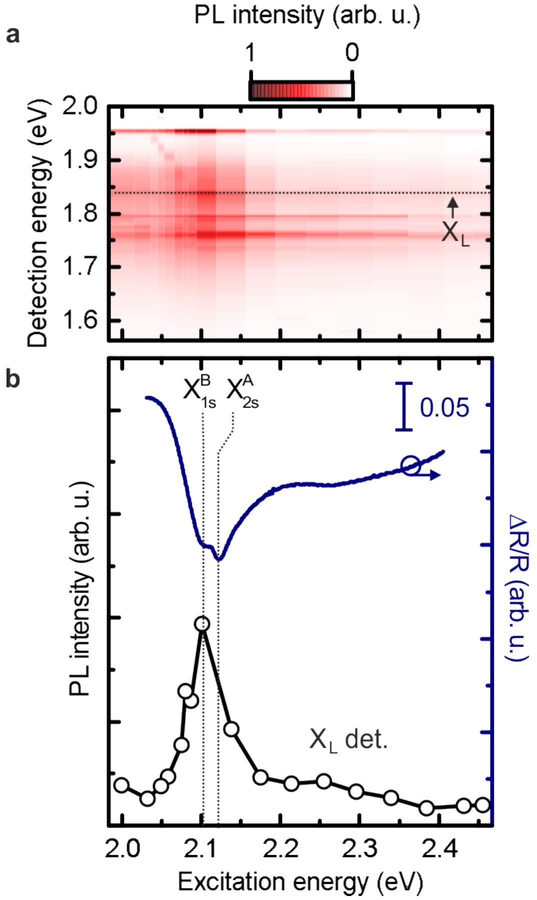

Supplementary Figure 7. **Photoluminescence excitation spectroscopy of single defect emitters in a helium ion bombarded hBN/MoS$_2$/hBN van der Waals heterostructure.** **a**, The false colour plot shows emission from $^0X^A_{1s}$ and localized emission X_L for excitation energetically above $^0X^A_{1s}$. **b**, Differential reflectivity $\Delta R/R$ of the heterostructure reveals the X^A_{2s} and X^B_{1s} as highlighted by the dashed lines. Photoluminescence intensity of X_L as a function of the laser excitation energy. The intensity enhancement of single defect emission and background coincides well when the laser is tuned on resonance with X^A_{2s} and X^B_{1s}.

Similar to the PLE measurements shown in Fig. 2. in the main manuscript, we performed additional measurements by scanning our excitation laser across excitonic resonances energetically above the $^0X^A_{1s}$ (cf. Supplementary Figure 7a). By measuring differential reflectivity $\Delta R/R$ using a broadband supercontinuum source, we observe several excitonic resonances as shown in Supplementary Figure 7b. The plot reveals absorption from the spin-orbit split $^0X^B_{1s}$ at an energy $E(^0X^B_{1s}) = 2.1\,\text{eV}$ and an additional resonance close to the $^0X^B_{1s}$ at

$E({}^0X_{2s}^A) = 2.12\,\mathrm{eV}$ which is ascribed to originate from the ${}^0X_{2s}^A$ as identified recently.[8] The hBN environment enhances optical quality and therefore allows us to observe higher excited Rydberg states.

Supplementary Note 8: He-ion exposed hBN prior to stacking of MoS_2

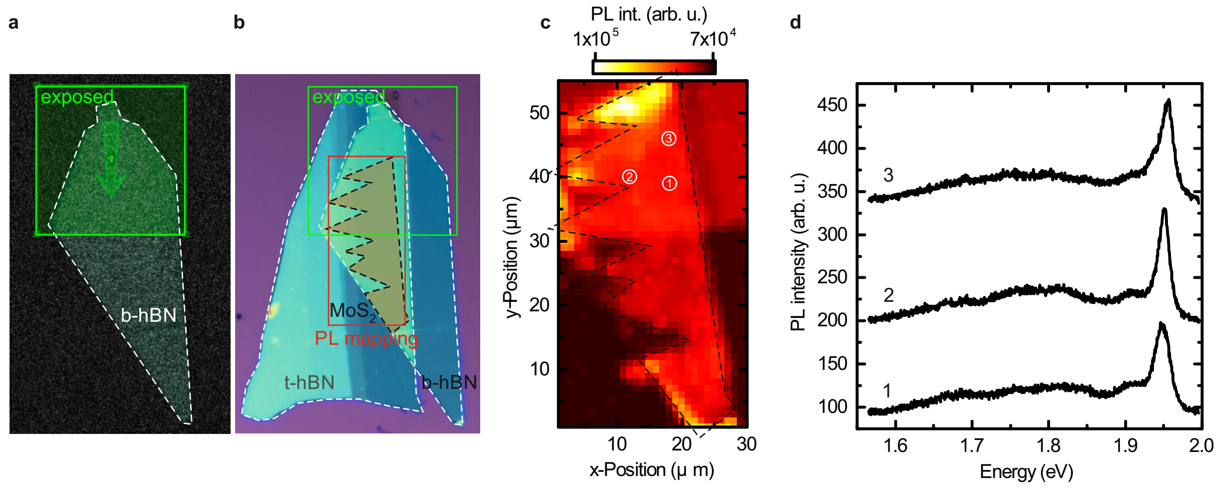

Supplementary Figure 8. **Spatially resolved photoluminescence of bombarded hBN with pristine MoS_2 and hBN stacked on top. a**, HIM image of the bottom hBN flake. The highlighted He-ion exposed area is treated with a dose of $\sigma = 2.2 \cdot 10^{12}\text{cm}^{-2}$. **b**, Optical microscope image of the hBN/MoS_2/hBN van der Waals heterostructure. **c**, Spatially resolved and spectrally integrated ($1.6 - 2.0\,\text{eV}$) μ-PL mapping of the highlighted area in **b**. **d**, Representative spectra of three different positions taken from the PL mapping in **c**.

In order to rule out any correlation of exposure induced photoluminesce with defects created in hBN through He-ion exposure we investigate van der Waals heterostructures where the bottom hBN flake is treated with He-ions. Supplementary Figure 8a depicts a HIM image of a hBN flake on a SiO_2 substrate. The highlighted area of the flake is exposed with He-ions with a dose of $\sigma = 2.2 \cdot 10^{12}\text{cm}^{-2}$. This dose has shown to result in a high density of single defect emitters (cf. Supplementary Figure 6). After the bombardment a single layer of MoS_2 is stacked onto the partially bombareded b-hBN and is in a subsequent step fully enapsulated with the t-hBN crystal. A spatially resolved and spectrally integrated μ-PL mapping of the highlighted area in fig 8b is presented in Supplementary Figure 8c. Representative spectra from three different positions as highlighted in Supplementary Figure 8c are shown in Supplementary Figure 8d. Here, the spectra reveal luminescence from the neutral and charged exciton as well as the L-peak, signified by the broad luminescence background at lower energies. Importantly, unlike bombarded and encapsulated MoS_2 as presented in

the main manuscript, the spectra from bombarded hBN do not feature any single defect emitters. We therefore can unambiguously exclude He-bombardment induced defects in hBN as a source of single defect emission.

Supplementary Note 9: Evolution of defect states from ab-initio computations

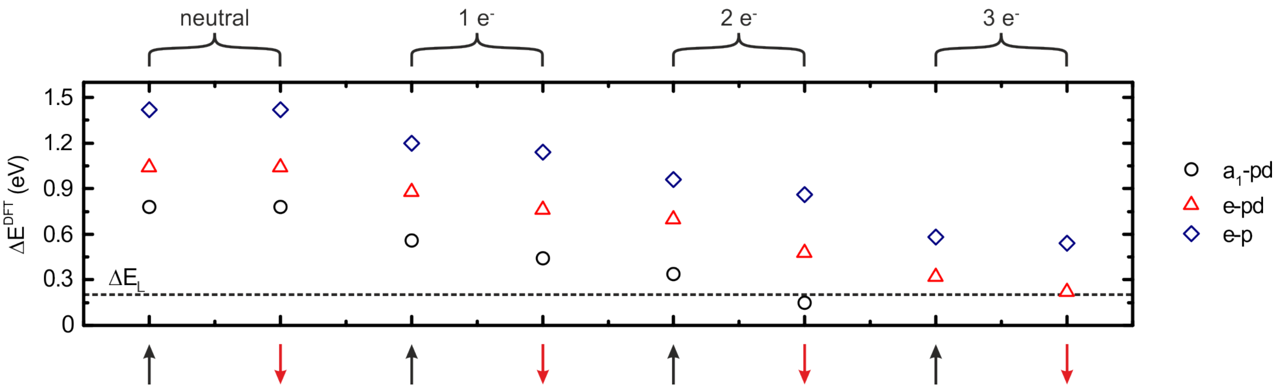

Supplementary Figure 9. **Energy difference of ingap defect states for differently charged molybdenum-vacancies.** Ab-initio computed energy differences $\Delta E^{DFT} = E(CBM)^{DFT} - E(state)^{DFT}$ are shown for a neutral V^0_{Mo} and for a single, double and triple charged molybdenum-vacancy (V^{1-}_{Mo}, V^{2-}_{Mo} and V^{3-}_{Mo}). Energy differences are shown for the *e-p*, *e-pd* and a_1-*pd* for both, spin down and spin up states. The experimentally obtained lowest emission energy of $\Delta E_L \sim 0.2\,\text{eV}$ is highlighted with a dashed line.

Supplementary Figure 9 presents the DFT calculated energy differences ΔE^{DFT} as taken from the DOS shown in Fig. 1f-i in the main manuscript. In particular, energy differences between the conduction band minimum (CBM) and the *e-p*, *e-pd* and a_1-*pd* spin up and spin down states are shown for a neutral V^0_{Mo} and for a single, double and triple charged molybdenum-vacancy (V^{1-}_{Mo}, V^{2-}_{Mo} and V^{3-}_{Mo}). Moreover, we highlight the experimentally observed highest energy detuning $\Delta E_L \sim 0.2\,\text{eV}$ in order to directly compare with theory. We find best agreement with the *e-pd* spin down state ($\Delta E^{DFT} \sim 0.22\,\text{eV}$) of the V^{3-}_{Mo} that also is the lowest unoccupied state for this charging configuration.

Supplementary Note 10: Activation energy of a single defect emitter

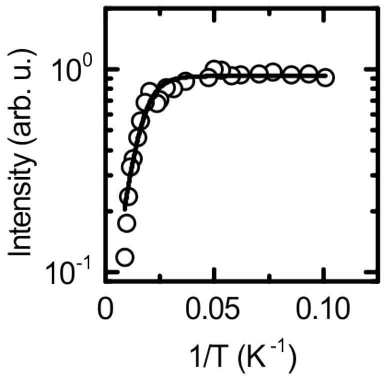

Supplementary Figure 10. **Activation energy of a single defect emitter.** Temperature dependent intensity of a single defect emitter X_L plotted in an Arrhenius representation. The black solid line is a fit with Eq. 1 to the data. The emitter exhibits an activation energy of (17.18 ± 2.05) meV.

From the temperature dependent data shown in Fig. 4a in the main manuscript, we also investigate the temperature dependent intensity of a single defect emitter X_L. The corresponding data is presented in Supplementary Figure 10 in an Arrhenius plot. The data is fitted by

$$I(T) = \frac{I_0}{1 + C \cdot \exp\left(-\frac{E_A}{k_B T}\right)}, \tag{1}$$

with the emission intensity at 0 K, a fitting constant C and the activation energy E_A. From the fit, we obtain an activation energy of (17.18 ± 2.05) meV. The activation energy is lower compared to the thermal activation energy $k_B T \sim 25\,\text{meV}$ at room temperature. This explains why emission is absent at elevated temperatures.

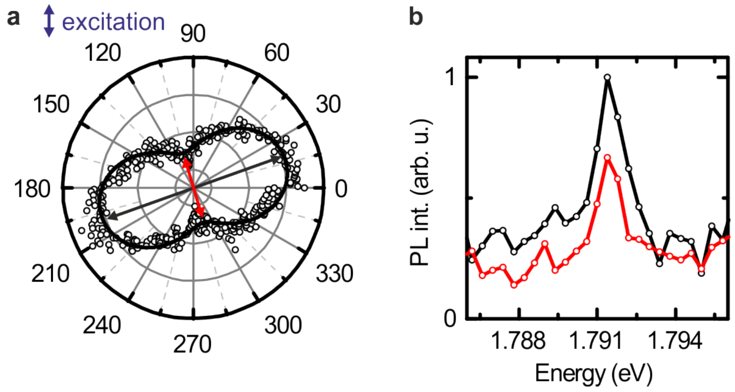

Supplementary Figure 11. **Dipolar emission characteristics of a single defect emitter.** **a**, Polar plot of the emission polarization characteristics of a single defect emitter for excitation with a fixed linear polarization (blue). Data are fitted with $\cos^2\theta$. **b**, Corresponding spectra for co- (black) and cross-linearly (red) detection polarization as highlighted by the arrows in **a**.

Supplementary Note 11: Emission polarization of a single defect emitter

To further support the interpretation that localized emission originates from a single dipole, we perform polarization resolved PL spectroscopy on a typical emitter at $\Delta E \sim 170\,\text{meV}$. We excite with a fixed linear excitation polarization and probe the emission polarization by rotating an analyzer in the detection path. The corresponding emission characteristic is shown in Supplementary Figure 11a. Two spectra for emission co- and cross-linearly aligned with the emission polarization are shown in Supplementary Figure 11b. This single photoluminescence line X_L exhibits a dipolar emission pattern, while the ${}^0X^A_{1s}$ emits isotropically. In turn, the former observation provides direct evidence that the luminescence X_L indeed originates from a single dipole. [9]

Supplementary Note 12: Defect emitter lineshape

To describe the defect emitter spectrum we utilize the independent boson model that has been succcessfully applied to describe the lineshape of quantum dot states [10–12] and defect-bound excitons [13]. The Hamiltonian for localized excitons of energy E coupled to lattice vibrations reads

$$H = EX^\dagger X + \sum_{\mathbf{q}} \hbar\omega_{\mathbf{q}} a^\dagger_{\mathbf{q}} a_{\mathbf{q}} + X^\dagger X \sum_{\mathbf{q}} g^X_{\mathbf{q}} (a^\dagger_{\mathbf{q}} + a_{-\mathbf{q}}) \,, \tag{2}$$

where the interaction part represents lattice deformations due to the presence of an exciton. The exciton-phonon matrix elements

$$g^X_{\mathbf{q}} = \sum_{\mathbf{k}} \phi^*(\mathbf{k}) \left[g^c_{\mathbf{q}} \phi(\mathbf{k} + \mathbf{q}_h) - g^v_{\mathbf{q}} \phi(\mathbf{k} - \mathbf{q}_e) \right] \tag{3}$$

contain the exciton wave function $\phi(\mathbf{k})$ with $\mathbf{q}_{\mathrm{e/h}}$ been the electron/hole momentum. For a localized s-exciton we obtain

$$g^X_{\mathbf{q}} = \frac{g^c_{\mathbf{q}}}{\left[1 + \frac{(a_{\mathrm{B}}|\mathbf{q}_h|)^2}{4}\right]^2} - \frac{g^v_{\mathbf{q}}}{\left[1 + \frac{(a_{\mathrm{B}}|\mathbf{q}_e|)^2}{4}\right]^2} \tag{4}$$

where a_{B} is the 2D exciton bohr radius. The carrier-phonon matrix elements $g^{c/v}$ are treated in deformation potential approximation [14, 15] and we account for the coupling with LA and TA phonon modes, which are found to be the dominant source of phonon dephasing in the system. For deformation potential coupling with acoustic phonons of dispersion $\omega_{\mathbf{q}} = v_s q$ the carrier-phonon matrix element is given by

$$g^{c/v}_{\mathbf{q}} = D^{c/v} \sqrt{\frac{\hbar\omega_{\mathbf{q}}}{2 v_s^2 \rho A}} \tag{5}$$

with sound velocity v_s, 2D mass density ρ, and deformation potentials $D^{c/v}$ taken from DFT/DFPT calculations [15, 16]. Here, A is the crystal area.

The spectral properties are obtained from the optical response to a classical electric field $E(t)$, which drives a polarization of the medium. In response to a weak optical (test) field, the linear optical susceptibility is given by:

$$\chi(\omega) = \frac{P(\omega)}{\varepsilon_0 E(\omega)} \,. \tag{6}$$

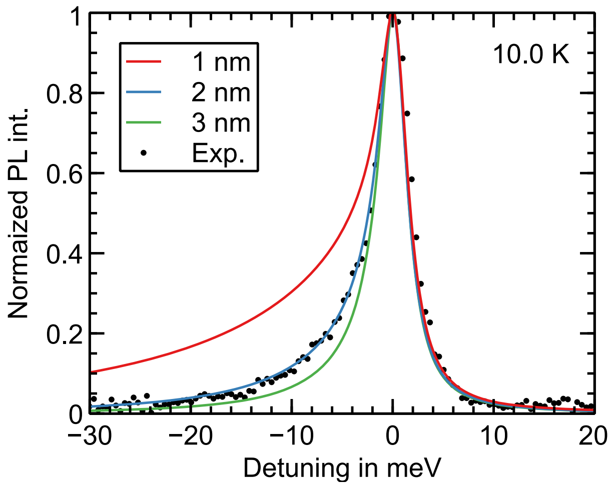

Supplementary Figure 12. **Comparison between measured and calculated emission spectra for various Bohr radii.** The result for $a_\mathrm{B} = 2\,\mathrm{nm}$ corresponds to the spectrum at 10 K in Fig. 3 of the main text.

considering the polarization P in direction of the external field. To obtain the frequency-dependent response of the medium, we use the Fourier transform of the polarization $P(t)$, which can be written for a delta-like excitation as [10]

$$P(t) = -i\Theta(t)\exp\left[-it(E - i\gamma_\mathrm{rad}) + R(t) - R(0) - tR'(0)\right]. \tag{7}$$

The polarization decays due to radiative recombination with a rate γ_rad as well as excitations into phonon sidebands described by the complex function

$$R(t) = \sum_\mathbf{q} \left|g^X_\mathbf{q}\right|^2 \left[\frac{N_\mathbf{q}}{(\omega_\mathbf{q} + i\gamma_\mathrm{ph})^2} e^{(+i\omega_\mathbf{q} - \gamma_\mathrm{ph})t} + \frac{N_\mathbf{q} + 1}{(\omega_\mathbf{q} - i\gamma_\mathrm{ph})^2} e^{(-i\omega_\mathbf{q} - \gamma_\mathrm{ph})t}\right] \tag{8}$$

where $N_\mathbf{q} = 1/(\exp[\hbar\omega_\mathbf{q}/k_B T] - 1)$ is the phonon occupation, $g^X_\mathbf{q}$ the exciton-phonon matrix elements, γ_{ph} the phonon lifetime and ω_q the acoustic phonon dispersion assumed in equilibrium at lattice temperature T. Absorption spectra that are obtained from solving Eqs. (6)-(8) posses a Lorentz-broadend zero-phonon line as well as phonon sidebands due to multi-phonon processes. The latter give rise to the observed asymmetric lineshape at low temperature, where only phonon emission processes dominate.

Emission spectra are obtained as a mirror image of the absorption spectra reflected across the zero-phonon line [17]. At low temperature best agreement between calculated and measured photoluminescence spectra is obtained for a Bohr radius of 2 nm, see Supplementary

Figure 12. In order to obtain this agreement, it was necessary to reduce the exciton-phonon coupling strength by a factor of 3.75 compared to the bare monolayer. This results in a Huang-Rhys factor

$$S = \sum_{\mathbf{q}} \frac{\left|g_{\mathbf{q}}^{X}\right|^2}{\omega_{\mathbf{q}}^2} \tag{9}$$

of 0.75, which is consistent with the phonon coupling that we have obtained from the analysis of the temperature dependent peak position (cf. main text). For the phonon lifetime $1/\gamma_{\mathrm{ph}}$ we used a value of 40 ps according to Ref. [18]. From the fit we obtain a value for the radiative linewidth of 0.5 meV. Using this parameters, all spectra in Fig. 3 of the main text are then well described by changing the temperature according to the experimental condition.

Supplementary references

---

[1] Tran, T. T.; Bray, K.; Ford, M. J.; Toth, M.; Aharonovich, I. Quantum emission from hexagonal boron nitride monolayers. *Nature Nanotechnology* **2015**, *11*, 37–41.

[2] Klein, J.; Kuc, A.; Nolinder, A.; Altzschner, M.; Wierzbowski, J.; Sigger, F.; Kreupl, F.; Finley, J. J.; Wurstbauer, U.; Holleitner, A. W.; Kaniber, M. Robust valley polarization of helium ion modified atomically thin MoS2. *2D Materials* **2017**, *5*, 011007.

[3] Cadiz, F. et al. Ultra-low power threshold for laser induced changes in optical properties of 2D molybdenum dichalcogenides. *2D Materials* **2016**, *3*, 045008.

[4] Wierzbowski, J.; Klein, J.; Sigger, F.; Straubinger, C.; Kremser, M.; Taniguchi, T.; Watanabe, K.; Wurstbauer, U.; Holleitner, A. W.; Kaniber, M.; Müller, K.; Finley, J. J. Direct exciton emission from atomically thin transition metal dichalcogenide heterostructures near the lifetime limit. *Scientific Reports* **2017**, *7*.

[5] Cadiz, F. et al. Excitonic Linewidth Approaching the Homogeneous Limit in MoS2 -Based van der Waals Heterostructures. *Physical Review X* **2017**, *7*.

[6] Florian, M.; Hartmann, M.; Steinhoff, A.; Klein, J.; Holleitner, A. W.; Finley, J. J.; Wehling, T. O.; Kaniber, M.; Gies, C. The Dielectric Impact of Layer Distances on Exci-

ton and Trion Binding Energies in van der Waals Heterostructures. *Nano Letters* **2018**, *18*, 2725–2732.

[7] Ajayi, O. A.; Ardelean, J. V.; Shepard, G. D.; Wang, J.; Antony, A.; Taniguchi, T.; Watanabe, K.; Heinz, T. F.; Strauf, S.; Zhu, X.-Y.; Hone, J. C. Approaching the intrinsic photoluminescence linewidth in transition metal dichalcogenide monolayers. *2D Materials* **2017**, *4*, 031011.

[8] Robert, C.; Semina, M. A.; Cadiz, F.; Manca, M.; Courtade, E.; Taniguchi, T.; Watanabe, K.; Cai, H.; Tongay, S.; Lassagne, B.; Renucci, P.; Amand, T.; Marie, X.; Glazov, M. M.; Urbaszek, B. Optical spectroscopy of excited exciton states in MoS2 monolayers in van der Waals heterostructures. *Physical Review Materials* **2018**, *2*.

[9] Jungwirth, N. R.; Chang, H.-S.; Jiang, M.; Fuchs, G. D. Polarization Spectroscopy of Defect-Based Single Photon Sources in ZnO. *ACS Nano* **2015**, *10*, 1210–1215.

[10] Zimmermann, R.; Runge, E. Dephasing in quantum dots via electron-phonon interaction. *Proc. 26th ICPS, Edinburgh* **2002**,

[11] Wilson-Rae, I.; Imamoğlu, A. Quantum dot cavity-QED in the presence of strong electron-phonon interactions. *Phys. Rev. B* **2002**, *65*, 235311.

[12] Krummheuer, B.; Axt, V. M.; Kuhn, T. Theory of pure dephasing and the resulting absorption line shape in semiconductor quantum dots. *Phys. Rev. B* **2002**, *65*, 195313.

[13] Duke, C. B.; Mahan, G. D. Phonon-Broadened Impurity Spectra. I. Density of States. *Phys. Rev.* **1965**, *139*, A1965–A1982.

[14] Kaasbjerg, K.; Thygesen, K. S.; Jacobsen, K. W. Phonon-limited mobility in n-type single-layer MoS_2 from first principles. *Physical Review B* **2012**, *85*, 115317.

[15] Jin, Z.; Li, X.; Mullen, J. T.; Kim, K. W. Intrinsic transport properties of electrons and holes in monolayer transition-metal dichalcogenides. *Physical Review B* **2014**, *90*, 045422.

[16] Li, X.; Mullen, J. T.; Jin, Z.; Borysenko, K. M.; Buongiorno Nardelli, M.; Kim, K. W. Intrinsic electrical transport properties of monolayer silicene and MoS_2 from first principles. *Physical Review B* **2013**, *87*, 115418.

[17] Mahan, G. D. *Many Particle Physics*, 3rd ed.; Springer, 2000.

[18] Gu, X.; Li, B.; Yang, R. Layer thickness-dependent phonon properties and thermal conductivity of MoS2. *Journal of Applied Physics* **2016**, *119*, 085106.
